# RELATIONSHIP BETWEEN JOB STRESS AND SELF-CARE ON MIDDLE-AGED AND OLDER ADULT EMPLOYEE WELL-BEING

**DOI:** 10.1093/geroni/igae098.2376

**Published:** 2024-12-31

**Authors:** Laurel Mertz, Jennifer Smith

**Affiliations:** Mather Institute, Evanston, Illinois, United States; Mather Institute, Evanston, Illinois, United States

## Abstract

Given that workplace stress and burnout are related to numerous adverse well-being outcomes, identifying strategies to decrease workplace stress or mitigate its effects is vital. Self-care may be a resource to protect against the negative impact of job stress on employees’ health and well-being, but self-care research is limited in non-healthcare fields. Additionally, as the number of older adults in the workforce rises, there is an increasing need to understand and enhance older adult employees’ well-being. The purpose of this study was to assess whether members of the middle-age and older adult workforce are able to engage in as much self-care as they need, and to determine whether the ability to engage in enough self-care attenuates the relationship between job stress and well-being outcomes. A total of 2,112 full-time employees, ages 43 to 77, were recruited via an online research panel (mean age = 54.0 years, 59.0% male, 64.2% White). Participants completed a survey that assessed job stress, ability to engage in self-care, and well-being (i.e. life satisfaction, job satisfaction, mental and physical health). Multiple regression analyses indicated that engaging in enough self-care attenuated the negative relationship between job stress and satisfaction with one’s life and job, but not health. These results provide evidence that self-care may be effective in protecting against the negative impact of workplace stress in the middle-age to older adult workforce. Further research is needed to examine the effectiveness of formal workplace self-care interventions, as formal interventions may supplement employees’ personal levels of self-care.

